# Silencing LncRNA SNHG16 suppresses the diabetic inflammatory response by targeting the miR-212-3p/NF-κB signaling pathway

**DOI:** 10.1186/s13098-023-01070-5

**Published:** 2023-06-07

**Authors:** Linjuan Huang, Shengxi Xiong, Hanshuang Liu, Ranran Zhang, Ying Wu, Xiaolei Hu

**Affiliations:** The Department of Endocrinology, The First Affiliated Hospital of Bengbu Medical College, Bengbu, 233000 China

**Keywords:** LncRNA, SNHG16, miR-212-3p, Diabetes, Inflammation

## Abstract

**Background:**

Long noncoding RNAs (LncRNAs) have been identified to play an important role in diabetes. The aim of the present study was to determine the expression and function of small nucleolar RNA host gene 16 (SNHG16) in diabetic inflammation.

**Methods:**

For the in vitro experiments, quantitative real-time PCR (qRT-PCR), Western blotting and immunofluorescence were used to detect LncRNA SNHG16 expression in the high-glucose state. The potential microRNA sponge target of LncRNA SNHG16, miR-212-3p, was detected by dual-luciferase reporter analysis and qRT-PCR. For the in vivo experiments, glucose changes in mice were detected after si-SNHG16 treatment, and SNHG16 and inflammatory factor expression in kidney tissues were detected by qRT-PCR and immunohistochemistry.

**Results:**

LncRNA SNHG16 was upregulated in diabetic patients, HG-induced THP-1 cells, and diabetic mice. Silencing SNHG16 inhibited the diabetic inflammatory response and the development of diabetic nephropathy. miR-212-3p was found to be directly dependent on LncRNA SNHG16. miR-212-3p could inhibitor P65 phosphorylation in THP-1 cells. The miR-212-3p inhibitor reversed the action of si-SNHG16 in THP-1 cells and induced an inflammatory response in THP-1 cells. LncRNA SNHG16 was also found to be higher in the peripheral blood of diabetic patients than in the normal person. The area under the ROC curve is 0.813.

**Conclusion:**

These data suggested that silencing LncRNA SNHG16 suppresses diabetic inflammatory responses by competitively binding miR-212-3p to regulate NF-κB. LncRNA SNHG16 can be used as a novel biomarker for patients with type 2 diabetes.

**Supplementary Information:**

The online version contains supplementary material available at 10.1186/s13098-023-01070-5.

## Introduction

Diabetes is a non-communicable disease characterized by oxidative stress and inflammation, the incidence of which is increasing year by year [1]. Diabetic nephropathy is the most common microvascular complication of diabetes, and its pathogenesis is also an inflammatory response [2, 3]. Since the mechanisms that maintain low levels of inflammation in chronic diabetes are not fully elucidated, it is necessary to study the pathogenesis of diabetic inflammation.

Long noncoding RNAs (LncRNAs) are families of noncoding molecular transcripts over 200 nucleotides in length that have been identified as having an important functional role in a variety of human diseases [4]. Studies have reported that multiple LncRNAs are involved in the regulation of diabetes and the development of diabetes [5]. Notably, small nuclear RNA host gene 16 (SNHG16), a recently discovered LncRNA, is mainly associated with the development and progression of various malignancies [6, 7]. SNHG16 has been less studied in the field of diabetes mellitus, and the pathological basis of cancer is also an inflammatory response. Studies in recent years have found that SNHG16 induces proliferation and fibrosis of chylomicron cells in mice with diabetic nephropathy [8]. Another study has reported that SNHG16 exacerbates high glucose-induced foot cell injury in diabetic nephropathy [9]. Based on our previous findings, we extracted Peripheral blood mononuclear cells from type 2 diabetic patients and normal person, and after LncRNA microarray sequencing, we found that SNHG16 was upregulated in diabetic patients [10]. The pathological mechanism of diabetic nephropathy is also an inflammatory response. The mechanism of SNHG16 in diabetic inflammation has not been investigated in the current study. Therefore, further study of the mechanism of SNHG16’s role in diabetic inflammation is necessary. Elucidating the mechanism of SNHG16 in diabetic inflammation could provide new directions for the treatment of diabetes.

miRNAs, which are small noncoding RNAs of approximately 22 nucleotides in length, regulate various cellular processes [11]. A growing body of evidence suggests that dysregulated expression of miRNAs is associated with the pathogenesis of a variety of diseases, including type 2 diabetes mellitus (T2DM). LncRNAs main function in the cytoplasm is to affect downstream target genes through competitive binding of miRNAs [12]. Therefore, the status of miRNAs is particularly important. Studies have shown that miR-212-3p plays an important role in both sepsis [13] and rheumatoid arthritis [14]. A recent study showed that miR-212-3p attenuates neuroinflammation in Alzheimer’s disease rats by regulating the NLRP3/caspase-1 signaling pathway [15]. Interestingly, we found that all of these diseases share a common feature in that they are all inflammation-based diseases. Therefore, we considered whether SNHG16 could affect diabetic inflammation by competitively binding to miR-212-3p.

The development of inflammation requires the involvement of multiple inflammatory signaling pathways and specific proinflammatory cytokines, and studies have confirmed that one of the important causes of chronic inflammation in diabetes is the activation of the nuclear factor-κB (NF-κB) signaling pathway [16]. When cells are stimulated, IκBα is phosphorylated and subsequently ubiquitinated and degraded by the proteasome, releasing active NF-κB (P65/P50) into the nucleus to regulate the transcription of multiple target genes, including inflammatory factors (TNF-α, IL-1 β and IL-6) [17]. A recent study has demonstrated that upregulation of SNHG16 promotes diabetes-associated retinal vascular endothelial cell (RMEC) dysfunction by activating the NF-κB and PI3K/AKT pathways [18], but the exact mechanism remains unclear.

In the present study, we investigated the role of SNHG16 in regulating NF-κB via miR-212-3p in diabetes inflammation, providing new insights for the development of future diabetes therapies.

## Materials and methods

### Cell culture and model construction

THP-1 cells (RRID:CVCL_0006) were obtained from the cell bank of Bengbu Medical College and cultured in RPMI 1640 medium supplemented with 10% fetal bovine serum and 1% penicillin/streptomycin at 37 °C in a humidified atmosphere of 5% CO_2_. Routine medium changes were made for passaging, and log phase cells were utilized for experiments. THP-1 cells were induced to become macrophages with 50 ng/ml PMA for 48 h. For subsequent steps, THP-1-induced macrophages were cultured for 2 days using RPMI 1640 medium with glucose concentrations of 5.5 and 25.0 mmol/L for the normal control (NC) and high glucose (HG) groups. LPS (100 ng/ml) and IFN-γ (20 ng/ml) in RPMI 1640 culture medium were used to culture THP-1-induced macrophages for 2 days for the inflammatory (LPS) group.

### Clinical samples

Clinical whole blood samples from diabetic patients and healthy individuals were collected from the Endocrine Laboratory of the First Affiliated Hospital of Bengbu Medical College from June 2022 to December 2022. Diabetic patients were diagnosed by passing the diagnostic criteria of the glucose tolerance test. Oral glucose tolerance test(OGTT): 2 h postprandial blood glucose>11.1 mmol/L. Fasting blood glucose ≥ 7.0mmol/L. Patients that met the Guideline for the prevention and treatment of type 2 diabetes mellitus in China (2020 edition) [19]. The exclusion criteria were as follows: (1) those with tumors, severe cardiovascular and cerebrovascular pathologies; (2) those with other complications of diabetes; (3) those with hyperthyroidism and other diseases affecting glucose metabolism; (5) polycystic ovaries, pregnancy, etc. All experiments were approved by the Medical Ethics Committee of Bengbu Medical College (Approval [2021] No. 210).

### Single nucleus cell extraction

An equal volume of lymphocyte isolate as whole blood was added to a 15 ml centrifuge tube, and fresh whole blood to which anticoagulant had been added was placed above the surface of the lymphocyte isolate and centrifuged at 20 °C for 30 min at 3000 g. The leukocyte layer was separated, washed with PBS and centrifuged to recover leukocytes.

### Cell transfection

The si-SNHG16 (homo), miR-212-3p mimics, miR-212-3p inhibitors and negative controls (siNC, mimic NC, and inhibitor NC) were purchased from General Biology. When THP-1 cells reached 70% confluency, gene transfection was performed using Lipofectamine 8000 transfection reagent according to the manufacturer’s protocol. si-SNHG16 (Mus) was purchased from Jiman Biotechnology.

### RNA extraction and quantitative real-time PCR (qRT-PCR)

Total RNA was extracted and reverse transcribed into cDNA using the Abscript II cDNA First-Strand Synthesis Kit Reverse transcriptase. Gene expression levels were determined in each group using 2×Universal SYBR Green Fast qPCR Mix and specific primers. PCR was performed in triplicate with the following thermocycler profile: denaturation at 95 °C for 10 min; 95 °C, 5s and 60 °C, 34 s for 40 cycles of amplification. GAPDH was used as the internal reference gene. The expression levels of mRNA and LncRNA were analyzed by the 2^−ΔΔCT^ method. For miR-212-3p, reverse transcription was performed using the miRNA 1st Strand cDNA Synthesis Kit, and qPCR was performed using the MiRNA Universal SYBR qPCR Master Mix kit. U6 snRNA was used as an endogenous control. qRT-PCR was performed on Applied Biosystems 7500 Real-Time PCR System (RRID:SCR_018051).

### Luciferase assay

For the luciferase reporter analysis, the 3’UTR of SNHG16 was cloned into the pmirGLO reporter construct to generate the SNHG16 wild type (WT) plasmid, and a SNHG16 mutant (Mut) reporter plasmid was also generated. The h-SNHG16-3UTR and miR-212-3p or negative control (NC) plasmids were cotransfected into 293-T cells using Lipofectamine 2000. After 48 h of transfection, luciferase activity was observed by a dual-luciferase reporter system.

### Western blot analysis

Total proteins from kidney samples and THP-1 cells were lysed and extracted with RIPA lysis buffer. After quantification using the BCA Protein Assay Kit, protein samples were separated by SDS-PAGE, transferred to PVDF membranes and blocked with 5% BSA. The PVDF membranes were incubated with primary antibodies overnight at 4 °C, and the PVDF membranes were incubated with HRP-labeled secondary antibodies for 1 h at room temperature. Antibodies P-P65 (Affinity Biosciences Cat# AF2006, RRID:AB_2834435) and IL-1β (ABclonal Cat# A19635, RRID:AB_2862708) and P65 (ABclonal Cat# A19653, RRID:AB_2862717) were diluted according to the manufacturer’s instructions. The membranes were developed using an ECL developer.

### ELISA

The culture medium was collected after 48 h of cell culture. Samples were centrifuged at 3500 r/min for 10 min, and the concentrations of TNF-α, IL-6 and IL-1β in the serum were measured using ELISA kits (WELLBIO, Cat#EH10497M, Cat#EH10293M, Cat#EH10269M).

### Immunofluorescence

To explore the intranuclear expression level of NF-κB, cellular immunofluorescence analysis was performed. THP-1 cells were fixed with 4% paraformaldehyde for 30 min, permeabilized with 0.1% Triton X-100 for 15 min and blocked with 5% BSA for 1 h. The cells were then incubated with primary antibodies against P-P65 (Affinity Biosciences Cat# AF2006, RRID:AB_2834435) and IL-1β (ABclonal Cat# A19635, RRID:AB_2862708) overnight at 4 °C followed by incubation with appropriate secondary antibodies protected from light for 1 h. DAPI was used for nuclear staining of the cells. Finally, the cells were observed using Olympus IX70 Inverted Fluorescence Microscope (RRID:SCR_018604).

### Animals

In total, 24 6-week-old male C57BL/6J mice (RRID:IMSR_JAX:000664) were used for the experiments. Four mice were housed in each cage, and the mice were maintained at a controlled temperature (22 ± 2 °C), humidity (50 ± 5%) and a 12 h light-dark cycle.

### Establishment of a mouse model of type 2 diabetes mellitus

After 1 week of acclimatization feeding, 18 mice were randomly assigned to a diabetic diet (67% basal diet + 10% lard + 20% sucrose + 2.5% cholesterol + 0.5% sodium cholate), while the nondiabetic control group (CON) was fed a basal diet. Food and water were provided ad libitum. After four weeks, the animals were divided into two groups as follows: normal control (n = 6 per group) and diabetic (n = 18 per group).

T2DM was induced by intraperitoneal injection of 60 mg/kg streptozotocin dissolved in citrate buffer (pH 4.5) once a week for two weeks. Nondiabetic mice were injected with citrate buffer only. Each week after the second injection, fasting blood glucose (FBG) was measured from the tail vein of mice using a one-touch selective glucometer. Mice with FBG > 11.1 mmol/L at least twice in four weeks were considered diabetic. After successful modeling of the diabetic model, the control group was fed a control diet, and the diabetic group continued to be fed a diabetic diet.

### Silencing of SNHG16 in mice

After 4 weeks, the diabetic group was randomly divided into the following three groups: high glucose group (n = 6 per group), si-NC group (n = 6 per group) and si-SNHG16 group (n = 6 per group). The si-NC group was injected with si-NC (2.5 nmol, 125 µl) in the tail vein, and the si SNHG16 group was injected with si-SNHG16 (2.5 nmol, 125 µl) in the tail vein. The control and high glucose groups were injected with equal amounts of saline. Fasting blood glucose was measured and recorded after three days. During the experiment, body weight, food intake, water intake and FBG levels were measured weekly. The animals were anesthetized at the end of the experiment. Blood samples were collected and centrifuged at 3000 × rpm for 15 min. Kidneys and livers were immediately frozen in liquid nitrogen and stored at -80 °C until they were used for analysis. All experiments were approved by the Medical Ethics Committee of Bengbu Medical College (Approval [2021] No. 265).

### Hematoxylin and eosin (H&E), Masson and Periodic Acid Schiff(PAS)staining

Paraffin-embedded kidney tissues were sectioned (4 μm), and the tissue sections were subjected to HE staining, Masson’s staining, and PAS staining. After dehydration and sealing, the pathomorphology of the kidney tissue was observed by light microscopy.

### Immunohistochemistry

Tissue samples were fixed, dehydrated, embedded in paraffin and sectioned. Immunohistochemical staining was performed using a p-P65 antibody (Affinity Biosciences Cat# AF2006, RRID:AB_2834435). The collagen content was also quantified by ImageJ (RRID:SCR_003070).

Statistical analysis.

The experimental results were statistically analysed using SPSS 22.0 statistical software. For the measurement data, the data were normally distributed as well as when the homogeneity of variance, and the result was expressed as the mean ± standard deviation ($\stackrel{-}{x}$ ± s). Independent samples t-test was used for comparison between two groups and one-way analysis of variance (ANOVA) was used to compare differences between groups, For skewed distribution or heterogeneity of variance, nonnormally distributed data were statistically analyzed using a nonparametric test and the results were expressed by Z. Test level α = 0.05. *P < 0.05, **P < 0.01 and ***P < 0.001 were considered statistically significant.

**Table 1:** PCR primer sequences (Homo sapiens)

GeneName	Primer sequences (forward)	Primer sequences (reverse)
SNHG16	CAGAATGCCATGGTTTCCCC	TGGCAAGAGACTTCCTGAGG
TNF-α	GATCAATCGGCCCGACTATC	TCCTCACAGGGCAATGATCC
IL-1β	TTCGACACATGGGATAACGAGG	TTTTTGCTGTGAGTCCCGGAG
IL-6	TTCGGCAAATGTAGCATG	AATAGTGTCCTAACGCTCATAC
NF-κB(P65)	GTGGGGACTACGACCTGAATG	GGGGCACGATTGTCAAAGATG
GAPDH	TCTGGCACCACACCTTCTA	TCTGGCACCACACCTTCTA
miR-212-3p	GCGCGTGAGGTAGTAGGTTGT	TAACAGTCTCCAGTCACGGC
U6	CCTGCTTCGGCAGCACA	TGGAACGCTTCACGAA

**Table 2:** PCR primer sequences (Mus musculus)

Gene Name	Primer sequences (forward)	Primer sequences (reverse)
SNHG16	TCCTCCTCCTTGGGTGCTCT	CCTTACATCCCTGCCTCCTCTA
TNF-α	CCGATGGGTTGTACCTTGTC	TGGAAGACTCCTCCCAGGTA
IL-1β	GCCACCTTTTGACAGTGATGAG	AAGGTCCACGGGAAAGACAC
IL-6	TGCAAGAGACTTCCATCCAG	TCCACGATTTCCCAGAGAAC
NF-κB(P65)	CCGGGATGGCTACTATGAGG	GGTCTCGCTTCTTCACACAC
GAPDH	CCCACTAACATCAAATGGGG	CCTTCCACAATGCCAAAGTT

**Table 3:** Sequences used for cell transfection (Homo sapiens)

Gene Name	Sequence (5ʹ→3ʹ)	Sequence (5ʹ→3ʹ)
si-SNHG16	CUUAAAACCACUUACAAUAtt	UAUUGUAAGUGGUUUUAAGtt
si-SNHG16 NC	UUCUCCGAACGUGUCACGUtt	ACGUGACACGUUCGGAGAAtt
miR-212-3p mimic	UAACAGUCUCCAGUCACGGCCtt	GGCCGUGACUGGAGACUGUUAtt
miR-212-3pmimic NC	UCACAACCUCCUAGAAAGAGUAGAtt	UCUACUCUUUCUAGGAGGUUGUGAtt
miR-212-3p inhibitor	GGCCGUGACUGGAGACUGUUAtt	
miR-212-3p inhibitor NC	UCUACUCUUUCUAGGAGGUUGUGAtt	

**Table 4:** Sequences used for cell transfection (Mus musculus)

Gene Name	Sequence (5ʹ→3ʹ)	Sequence (5ʹ→3ʹ)
si-SNHG16	CGGAAGCCUUUGACAGCUAtt	UAGCUGUCAAAGGCUUCCGtt
si-SNHG16 NC	UUCUCCGAACGUGUCACGUtt	ACGUGACACGUUCGGAGAAtt

## Results

The inflammatory response in diabetes is a major cause of diabetic kidney injury. Exploration of diabetic inflammation-related biomarkers and their potential mechanisms is necessary to enrich therapeutic approaches. In the present study, we found that SNHG16 was upregulated in the high-glucose state, and SNHG16 knockdown suppressed the inflammatory response in the high-glucose state (TNF-α, IL-1β and IL-6 were all downregulated). In addition, miR-212-3p was found to be a downstream molecule of SNHG16. Silencing SNHG16 inactivated the NF-κB pathway in THP-1 cells and attenuated renal injury in diabetic mice in vivo. SNHG16 interacted with miR-212-3p to regulate P65 expression. In rescue experiments, the miR-212-3p inhibitor reversed the effects of SNHG16 knockdown on the THP-1 cell inflammatory response and NF-κB expression. Finally, we validated at the clinical level and found that SNHG16 was higher in peripheral blood of diabetic patients than normal person. the area under the ROC curve was 0.813, which indicates the significance of SNHG16 for the diagnosis of diabetes mellitus. Overall, the present findings demonstrated that si-SNHG16 attenuates the high glucose-induced inflammatory response in diabetes through the miR-212-3p/NF-κB axis and SNHG16 can be used as a novel biomarker for patients with type 2 diabetes.

### Effects of SNHG16 on diabetic status

**Fig. 1:**
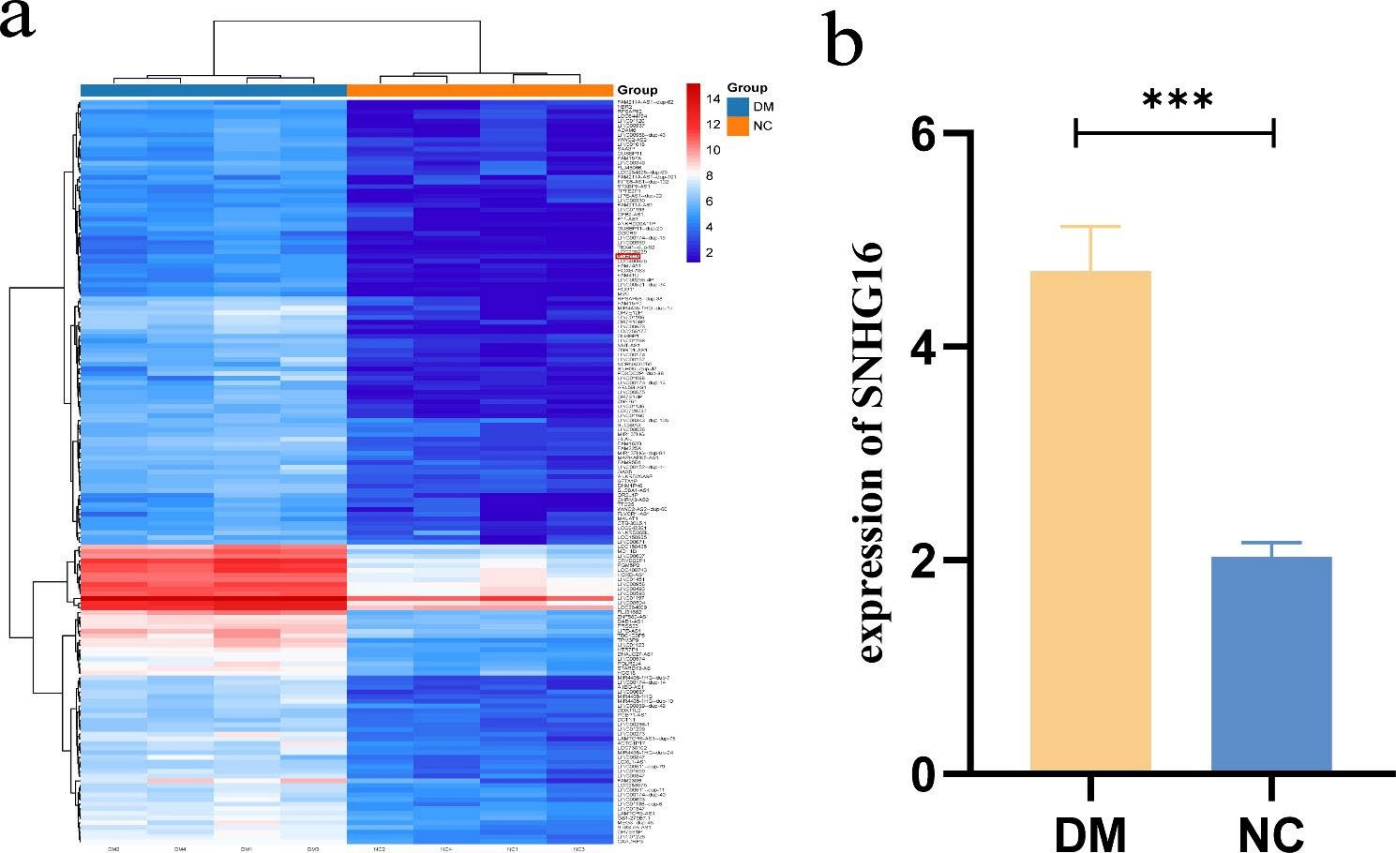
Heatmap and RNA clustering analysis of the top 100 genes (**a**). RNA microarray results for the expression of SNHG16 (**b**)

**Fig. 2:**
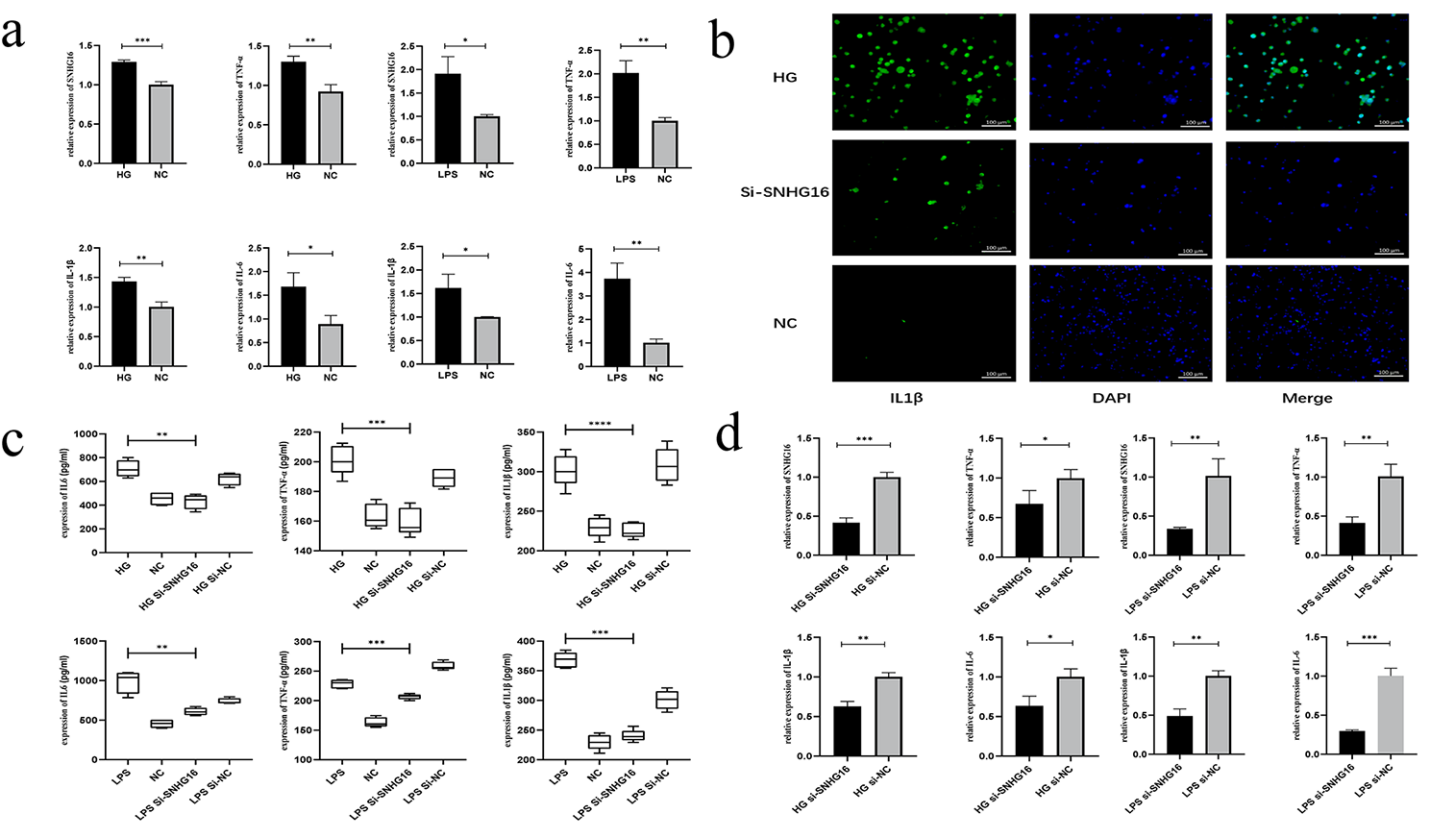
qRT-PCR analysis of HG and LPS-induced THP-1 cells. HG and LPS induced high expression of SNHG16 and inflammatory factors in THP-1 cells, whereas inflammatory factors were downregulated after si-SNHG16 transfection (**a, d**). Immunofluorescence analysis demonstrated that HG increased the IL-1β fluorescence intensity in THP-1 cells (**b**). ELISA demonstrated that HG and LPS upregulated the inflammatory factors in THP-1 cells, whereas the inflammatory factors were downregulated after si-SNHG16 treatment (**c**)

We performed a clustering analysis of the pre-RNA microarray results and found that the top 100 genes of the top variance genes contained SNHG16 (Fig. 1a), which suggested that SNHG16 was expressed at higher levels in diabetic patients than in healthy individuals (Fig. 1b). qRT-PCR further confirmed that SNHG16 was highly expressed in high glucose-induced THP-1 and LPS-induced THP-1 cells (Fig. 2a). We then measured inflammatory factors (TNF-α, IL-1β and IL-6) in both high glucose- and LPS-induced THP-1 cells compared to control cells (Fig. 2b-d). In addition, downregulation of si-SNHG16 in high glucose-induced and LPS-induced THP-1 cells reduced the levels of inflammatory factors (TNF-α, IL-1β and IL-6) (Fig. 2b-d).

### SNHG16 targets miR-212-3p and acts as a molecular sponge in THP-1 cells

The mechanism by which LncRNAs act as miRNA molecular sponges to bind miRNAs and thus indirectly regulate target genes is called the ceRNA network. To identify downstream miRNAs of SNHG16 in THP-1 cells, we used starBase prediction to identify potential binding sites of SNHG16 to miR-212-3p (https://starbase.sysu.edu.cn/index.php). The results suggested that SNHG16 represses miR-212-3p expression directly or indirectly. Therefore, we tested for a direct interaction between SNHG16 and miR-212-3p using a dual-luciferase reporter assay to determine the binding potential between the two RNAs.

**Fig. 3:**
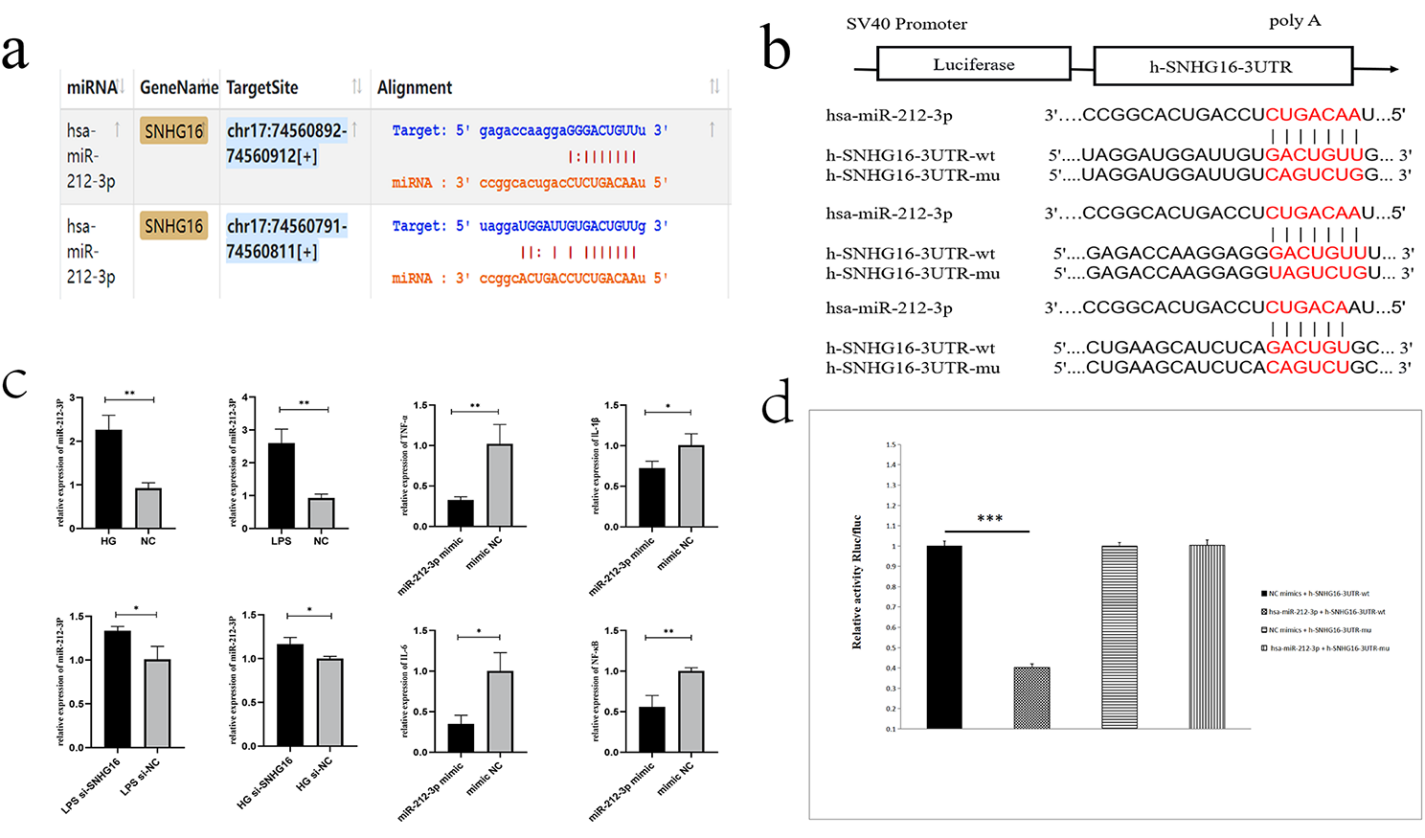
SNHG16 and miR-212-3p predicted binding sites (**a**). Schematic representation of SNHG16 mutant sites (**b**). qRT-PCR demonstrated that miR-212-3p was upregulated in the HG and LPS states but that inflammatory factors were downregulated after transfection of the miR-212-3p mimic (**c**). Dual-luciferase gene reporter assay (**d**)

Subsequently, a series of experiments were performed to validate this prediction. The results showed that miR-212-3p inhibited the luciferase activity of wild-type SNHG16, but the luciferase activity of mutant SNHG16 with three mutant binding sites was unaffected. In addition, mutant miR-212-3p failed to exhibit the aforementioned inhibitory effect (Fig. 3b, d). We found that the miR-212-3p mimic attenuated the inflammatory response in THP-1 cells, and SNHG16 silencing resulted in increased miR-212-3p levels in THP-1 cells (Fig. 3c). These results demonstrated that SNHG16 directly binds miR-212-3p in THP-1 cells.

### SNHG16 regulates P65 expression through competitive binding of miR-212-3p

**Fig. 4:**
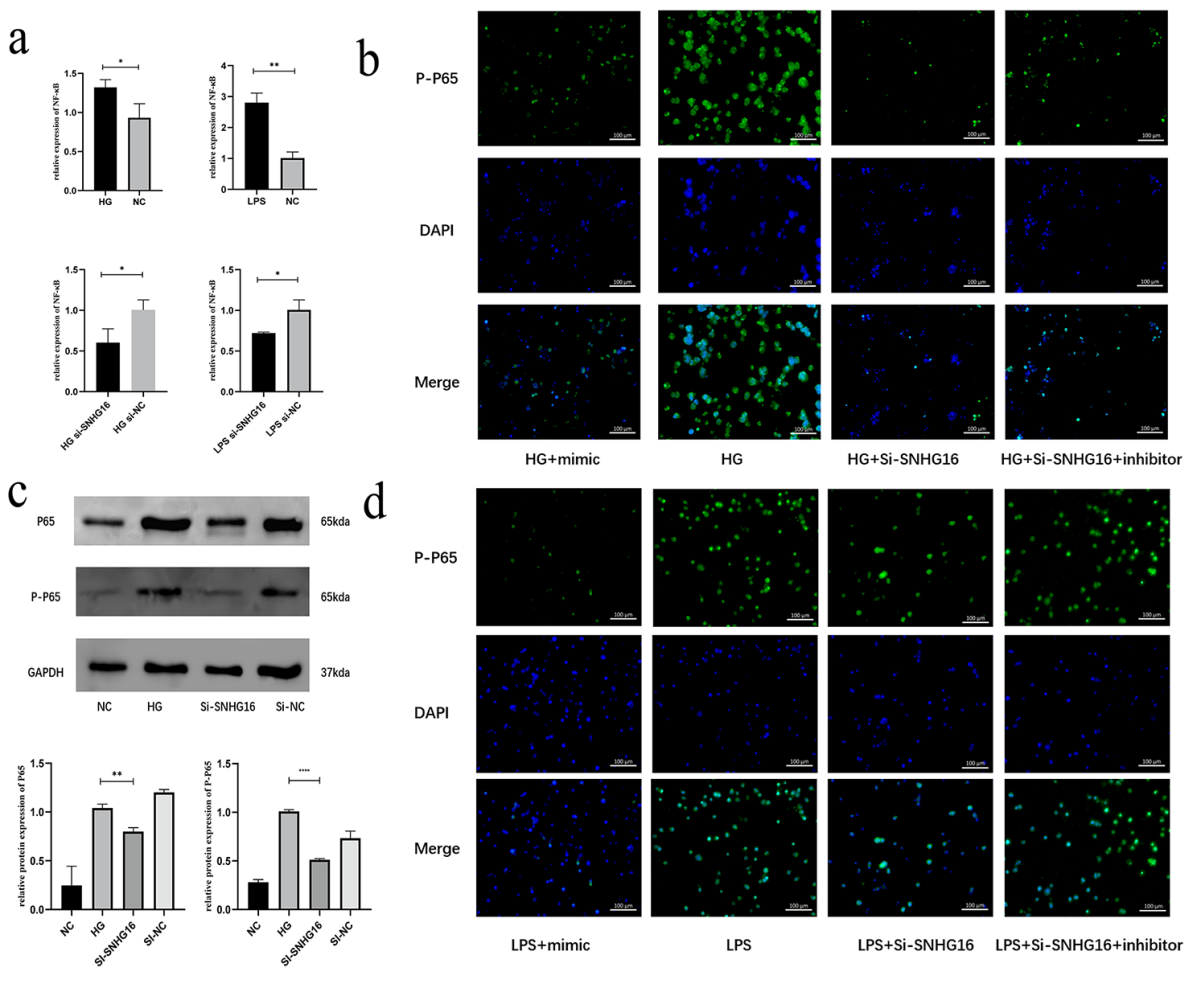
qRT-PCR analysis demonstrated that NF-κB expression was upregulated in HG and LPS states but was downregulated after si-SNHG16 transfection (**a**). Immunofluorescence analysis indicated that P-P65 expression was upregulated in HG and LPS states but was reduced after transfection of miR-212-3p mimic and si-SNHG16. However, treatment with the miR-212-3p inhibitor reversed this response (**b, d**). WB analysis indicated that P65 and P-P65 were upregulated in HG states but downregulated after si-SNHG16 (**c**)

To further elucidate the ceRNA network associated with SNHG16 and miR-212-3p, we investigated the specific target genes of miR-212-3p that regulate THP-1 under high glucose conditions, and we mainly focused on NF-κB, the predominant pathway of inflammation. We also predicted the target gene of NF-κB (P65) by miRsystem (http://mirsystem.cgm.ntu.edu.tw/) and found that miR-212-3P interacted with NF-κB (P65). In the HG and LPS states, NF-κB (P65) expression was upregulated, but NF-κB (P65) expression was downregulated after si-SNHG16 transfection (Fig. 4a-d). Western Blot (WB) showed that both P65 and P-P65 expression were down-regulated after silencing SNHG16 in HG state (Fig. 4c). In addition, P-P65 expression was downregulated after miR-212-3p mimic and si-SNHG16 transfection, but treatment with si-SNHG16 + miR-212-3p inhibitor reversed this response (Fig. 4b, d). These results suggested that SNHG16 regulates P65 expression through competitive binding of miR-212-3p.

### SNHG16 deficiency lowers blood glucose to reduce inflammation and diabetic kidney injury

**Fig. 5:**
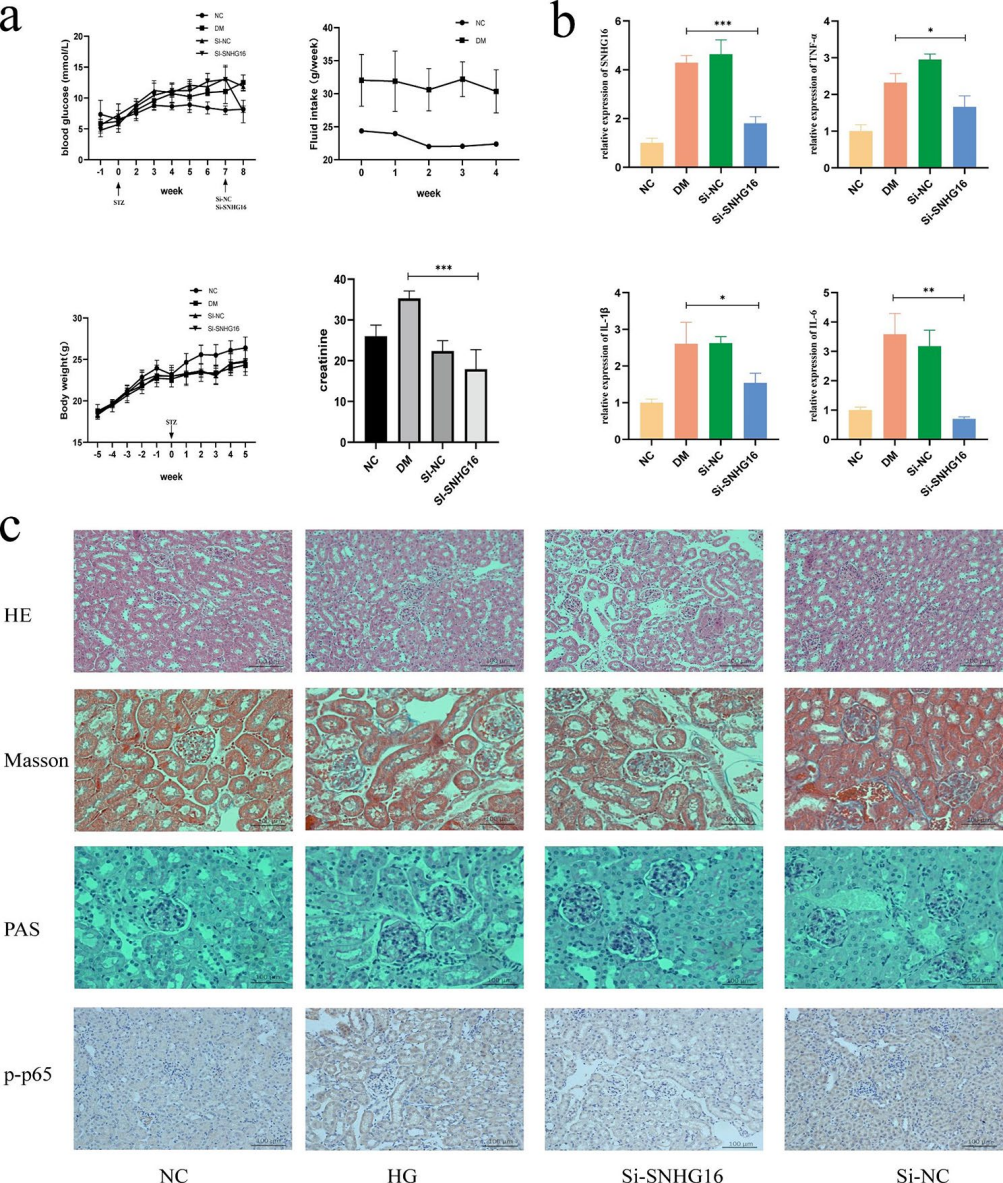
Diabetic and normal mice in general (**a**). qRT-PCR analysis of SNHG16 and inflammatory factor expression in renal tissues (**b**). HE staining, Masson’s staining and PAS staining as well as immunohistochemical fluorescence staining of P-P65 (**c**)

We investigated the effect of SNHG16 on diabetes-induced renal injury in vivo. Model mice with diabetic characteristics were obtained (Fig. 5a), indicating successful establishment of a diabetic model. We next measured the SNHG16 expression changes in the kidney tissue of diabetic mice, which demonstrated that SNHG16 was significantly increased in the kidneys of diabetic mice compared with NC mice (Fig. 5b). To assess the potential role of SNHG16 in protection against diabetic nephropathy, we evaluated changes in blood glucose in mice after si-SNHG16 treatment (Fig. 5a). SNHG16 inhibition in model mice reduced the concentrations of proinflammatory cytokines (TNF-α, IL-6 and IL-1β) (Fig. 5b), which suggested that SNHG16 silencing in diabetic mice attenuates diabetic kidney inflammation. In addition, knockdown of SNHG16 attenuated diabetic kidney injury as indicated by reduced creatinine levels in kidney tissue (Fig. 5a).

Further experiments were performed to assess the function of SNHG16 in diabetic kidney injury in diabetes. Pathological results showed significant glomerular hypertrophy, increased thylakoid cells, reduced cystic lumen, disorganized cystic wall structure and increased neutrophil exudation in the model group, and SNHG16 inhibition attenuated these effects (Fig. 5c). Immunohistochemical results showed that the P-P65 protein levels were reduced after SNHG16 silencing in vivo compared to the NC or si-NC group. These data suggested that SNHG16 deficiency attenuates diabetes-induced kidney injury.

Furthermore, knockdown of SNHG16 downregulated P65 expression as well as reduced P65 phosphorylation at Ser536 in diabetic kidney tissue (Fig. 5c), which suggested that SNHG16 regulated diabetic kidney tissue injury through P65 phosphorylation.

Overall, these results suggested that knockdown of SNHG16 exerts a protective effect on diabetic kidney tissue, which is partly due to alleviating the inflammatory response in the diabetic kidney.

### SNHG16 expression in diabetic patients

**Fig. 6:**
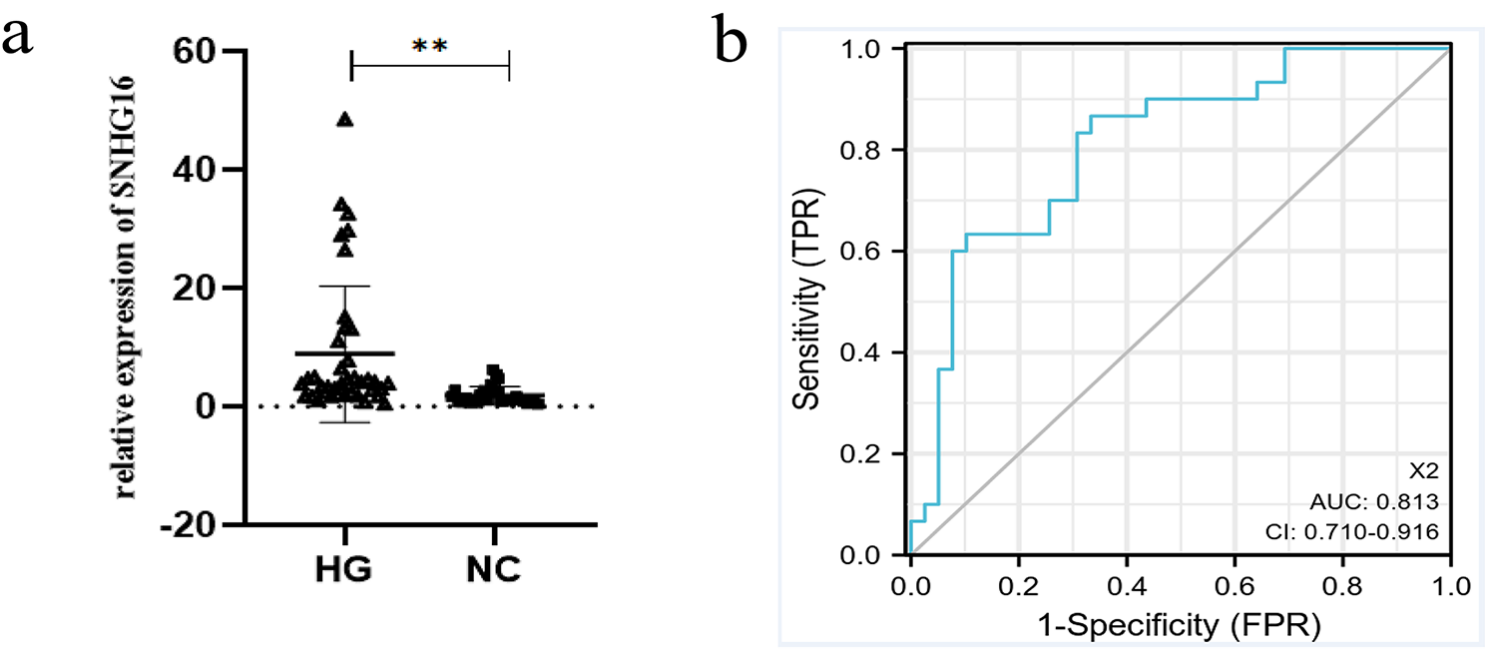
SNHG16 expression in diabetic and healthy individuals (**a**). ROC curve (**b**)

Finally, we collected whole blood from 30 healthy individuals and 39 diabetic patients for validation and extracted single nucleated cells. We compared general information between diabetic patients and healthy individuals (Table 5) and performed a binary logistic regression analysis (Table 6) on the factors influencing the occurrence of diabetes. qRT-PCR analysis demonstrated that the expression of SNHG16 was higher in diabetic patients than in healthy individuals (Fig. 6a). Logistic regression analysis found that both age and SNHG16 were risk factors for the development of diabetes mellitus. We generated an ROC curve to obtain the area under the curve (Fig. 6b), which indicated that SNHG16 may be a significant reference for the diagnosis of diabetes mellitus.

**Table 5:** Comparison of general information between the diabetic patients and healthy individuals

Group	Number	Gender	Age	BMI	FBG (mmol/L)	2hFBG	SNHG16
NC	30	2/28	33.23 ± 9.48	25.06 ± 4.47	5.10 ± 0.45	6.13 ± 0.86	1.91 ± 1.48
T2DM	39	20/19	46.74 ± 15.06	25.95 ± 4.17	7.65 ± 1.95	12.73 ± 4.72	8.87 ± 11.47
Z/t	-	0.69	-3.45	0.84	-6.64	-7.04	-4.43
P	-	<0.01	<0.01	0.401	<0.01	<0.01	<0.01

**Table 6:** Binary logistic regression analysis of factors influencing the occurrence of diabetes mellitus

Factors	B	Standard Error	*Wald χ* ^*2*^	*P*	OR	95% confidence interval
Age	-0.86	0.30	8.061	0.005	0.917	0.864–0.974
Sex	-1.716	0.916	3.508	0.061	0.180	0.039–1.083
BMI	-0.059	0.079	0.550	0.458	0.943	0.807–1.102
SNHG16	-0.584	0.242	5.141	0.023	0.578	0.360–0.928

Both age and SNHG16 were risk factors for diabetes. p < 0.05

**Fig. 7:**
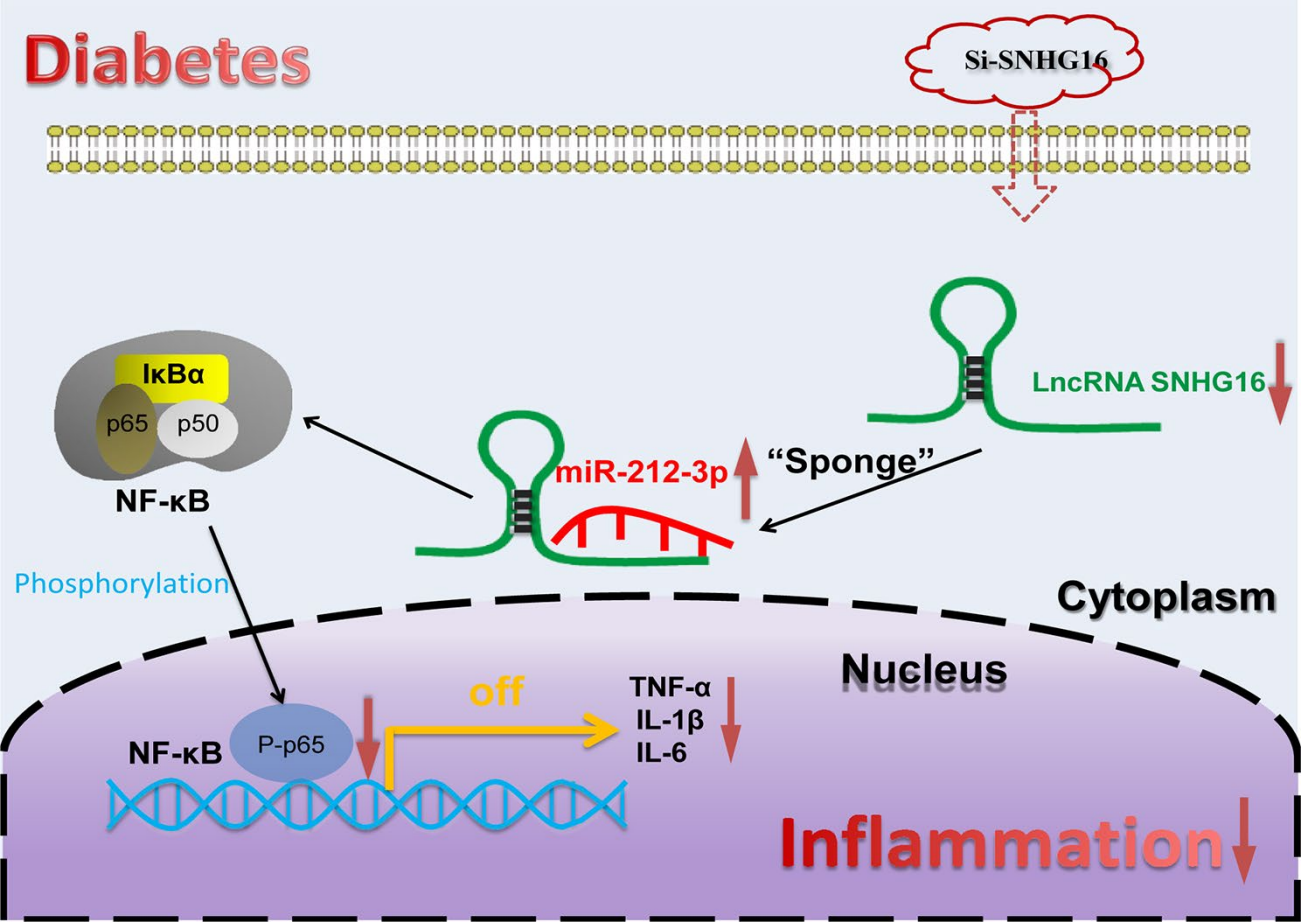
Diagram of the SNHG16 mechanism in diabetes

## Discussion

Diabetes mellitus is a chronic inflammatory disease, and it is associated with several life-limiting complications, including macrovascular and microvascular complications [20]. Diabetic nephropathy is one of the most common microvascular complications of diabetes and is a key site for the development of diabetic inflammation [21]. The present study investigated the mechanisms that may contribute to the development of diabetic inflammation and the potential factors that cause diabetic nephropathy, providing new insights into the treatment of diabetes.

LncRNAs are characterized as key regulators of gene expression at different levels, and they play important roles in a variety of biological processes [22]. In recent years, multiple LncRNAs have been found to be involved in the progression or prevention of diabetes. A previous study has reported that LncRNA Tug1 regulates the bioenergetic environment of patients with mitochondrial diabetic nephropathy [21]. Currently, SNHG16 is considered as an oncogene in many cancers [23, 24]. However, it has been less studied in diabetes. Recent studies have shown that SNHG16 exacerbates diabetic nephropathy through stabilizing TLR4/NF-κB pathway activation [25]. Furthermore, our previous LncRNA microarray results show that SNHG16 is highly expressed in diabetic patients [10]. Therefore, the aim of this study was to investigate the mechanism of the SNHG16 in regulating diabetic inflammation and diabetic nephropathy, providing new ideas for the treatment of diabetes.

To mimic the diabetic inflammatory state induced by hyperglycemia, we cultured THP-1 cells under HG conditions and used LPS induction as a positive control. Changes in SNHG16 and inflammatory factor expression were detected. We found that SNHG16 and inflammatory factors were significantly upregulated after HG exposure, suggesting that SNHG16 levels may be associated with the development of diabetic inflammation. Previous studies have found that SNHG16 upregulation can activate NF-kB and PI3K/AKT pathways to promote diabetes-associated RMEC dysfunction [18]. Thus, this study provides new evidence for the role of LncRNAs in diabetes. In addition, we also assessed the biological function of SNHG16, the effect of SNHG16 knockdown on diabetes and the reduction in inflammatory status after SNHG16 knockdown. Recent studies have shown that silencing SNHG16 inhibits the proliferation and inflammatory response of tuberculosis macrophages [26], which was consistent with the present findings.

We also explored the mechanism of SNHG16 in diabetes. The main mechanism of LncRNAs is through the competitive binding mechanism of miRNAs [27, 28], and LncRNA H19/miR-675 and LncRNA NEAT1/miR-204 have been reported to interact in a competitive binding manner in breast cancer [29]. Therefore, we searched for the downstream miRNA of SNHG16. By target gene prediction, we identified a miR-212-3p-binding site in SNHG16. In the present study, we verified the binding of SNHG16 to miR-212-3p by a dual-luciferase reporter gene assay. Previous studies have shown that miR-212-3p plays an important role in various cancers [30, 31], and other studies have reported that miR-212-3p inhibits macrophage inflammatory factor production [32]. One study found that Circ-Klhl8 overexpression increased the therapeutic effect of EPCs in diabetic wound healing through the miR-212-3p/SIRT5 axis [33]. Another study found that LncRNA XIST competitively binds hsa-miR-212-3p to regulate the expression of ASF1A in acute kidney injury [34]. However, miR-212-3p has not been reported in the literature in diabetic nephropathy and diabetic inflammation. Our study found that si-SNHG16 could upregulate miR-212-3p. miR-212-3p mimic was able to suppress the expression of inflammatory factors. Therefore, the investigation of the mechanism by which SNHG16 affects diabetic inflammation and diabetic nephropathy through competitive binding of miR-212-3p is also an innovative point of our study.

NF-κB, one of the key signaling pathways of inflammation, has been shown to be a key factor in the development of diabetic inflammation [1, 35, 36]. NF-κB has also been extensively studied in diabetic nephropathy [2, 37, 38]. In our study, we found that knockdown of SNHG16 attenuated kidney injury and decreased inflammatory factor expression in mouse kidney tissues. Recent studies have shown that SNHG16 exacerbates diabetic nephropathy by stabilizing TLR4 to regulate RAS and NF-κB pathway-mediated NLRP3 inflammatory vesicle activation [25]. This is consistent with the results of our study. We also found that both miR-212-3p mimics and si-SNHG16 could inhibit NF-κB (P65) expression, which was partially reversed by si-SNHG16 + miR-212-3p inhibitor. Through several in vitro and in vivo genetic assays, we found that SNHG16 reduced the inflammatory response in diabetes and diabetic kidney injury by competitively binding miR-212-3p to reduce the phosphorylation of P65.

Finally, we validated these findings in clinical patients. Importantly, the expression of LncRNA SNHG16 was higher in blood specimens from diabetic patients than in blood specimens from healthy individuals. ROC curve analysis indicated that LncRNA SNHG16 has diagnostic significance for the diagnosis of type 2 diabetes. Future studies should focus on the correlation between LncRNA SNHG16 and the clinical characteristics of diabetic patients, which may help to further define the clinical significance of LncRNA SNHG16.

## Conclusion

Overall, the present study showed that LncRNA SNHG16 is significantly upregulated in diabetic patients, high glucose-induced THP-1 cells, and diabetic mice. In addition, si-SNHG16 inhibits the diabetic inflammatory response as well as the development of diabetic nephropathy. Our findings revealed that LncRNA SNHG16 may regulate the diabetic inflammatory response by acting as a competitive sponge for miR-212-3p to regulate NF-κB 7. LncRNA SNHG16 may be used as a novel biological marker in patients with type 2 diabetes. Thus, these findings suggested that LncRNA SNHG16 may be developed as a new prognostic marker or therapeutic target that may help in the diagnosis and treatment of diabetes.

## Electronic supplementary material

Below is the link to the electronic supplementary material.

Supplementary Material 1

## Data Availability

The datasets generated during and/or analyzed during the present study are available from the corresponding author upon reasonable request.

## List of Abbreviations

LncRNAs: Long noncoding RNAs
NF-κB: Nuclear factor-κB
SNHG16: Small nuclear RNA host gene 16
T2DM: Type 2 diabetes mellitus
WT: Wild type
HG: High glucose
NC: Negative control
CON: Control group
