# Supplementary material for: Silencing LncRNA SNHG16 suppresses the diabetic inflammatory response by targeting the miR-212-3p/NF-κB signaling pathway
Source: Diabetol Metab Syndr. 2023 Jun 7;15:119. doi: 10.1186/s13098-023-01070-5 (PMC10245462; doi:10.1186/s13098-023-01070-5)
Supplement: Supplementary file 1 — Supplementary Material 1 [file 13098_2023_1070_MOESM1_ESM.pdf]

Editing Certificate

This document certifies that the manuscript

Silencing LncRNA SNHG16 suppresses diabetic inflammatory response by targeting miR-212-3p/ NF-κ B signaling pathway

prepared by the authors

Linjuan Huang, Xiaolei Hu1

was edited for proper English language, grammar, punctuation, spelling, and overall style by one or more of the highly qualified native English speaking editors at AJE.

This certificate was issued on **December 13, 2022** and may be verified on the AJE website using the verification code **6E92-9ACB-8D2B-1255-1433** .

Neither the research content nor the authors' intentions were altered in any way during the editing process. Documents receiving this certification should be English-ready for publication; however, the author has the ability to accept or reject our suggestions and changes. To verify the final AJE edited version, please visit our verification page at aje.com/certificate. If you have any questions or concerns about this edited document, please contact AJE at support@aje.com.

AJE provides a range of editing, translation, and manuscript services for researchers and publishers around the world.
For more information about our company, services, and partner discounts, please visit aje.com.
